# Understanding type 2 diabetes self-management in the Marshall Islands: A mixed-methods exploration of sociocultural and structural barriers

**DOI:** 10.1371/journal.pgph.0007152

**Published:** 2026-09-15

**Authors:** Jun Yoshino, Francyne Wase-Jacklick, Hilia Konou Enos, Ishael Ken, Hirotsugu Aiga, Ken Masuda

**Affiliations:** 1 School of Tropical Medicine and Global Health, Nagasaki University, Nagasaki, Japan; 2 Republic of the Marshall Islands Ministry of Health & Human Services, Majuro, Marshall Islands; Directorate of Factories, INDIA

## Abstract

Type 2 diabetes mellitus (T2DM) poses a critical public health challenge in the Republic of the Marshall Islands, where prevalence rates are among the highest globally. Despite the central role of self-management in diabetes care, adherence and utilization of health services remain inadequate and are major contributors to diabetes-related morbidity and mortality. We aimed to identify factors influencing self-management and to explore the sociocultural context shaping these behaviors using an explanatory sequential mixed methods approach. From March to May 2023, we surveyed 123 randomly selected T2DM outpatients aged 18 years and older in Majuro Atoll. We assessed self-management practices, illness perception, diabetes knowledge, clinical characteristics, and sociodemographic factors, and used regression analysis to identify key predictors of self-management. Subsequently, semi-structured interviews with ten patients and three healthcare providers were conducted and analyzed thematically. Findings revealed that 76% of participants demonstrated inadequate self-management. Despite 90% recognizing diet as a causative factor, dietary control scored lowest among self-management domains, highlighting a disconnect between awareness and action. A higher perceived threat of illness was associated with poorer self-management, suggesting that fear may discourage rather than encourage coping action. While attending diabetes education classes was positively associated, diabetes knowledge level was not a significant predictor. Three key themes emerged as barriers to self-management that lie beyond individual control: *i.Dysfunctional healthcare service delivery*, *ii. Diabetes fatalism*, and *iii. Food desert*. This study highlights the need for culturally tailored health education, psychological support to counter diabetes fatalism along with strengthening health systems to better respond to patient needs, and policies to improve food security, which are critical to improving diabetes outcomes in this high-risk population.

## 1. Introduction

Diabetes mellitus is widely regarded as a significant global health challenge, garnering attention within the field of public health [1]. The global prevalence of diabetes is escalating rapidly, with an estimated 529 million individuals affected as of 2021, constituting 6.1% of the global population. Projections indicate a further increase to 10.2% by the year 2030 [2,3]. While diabetes is classified into four types, Type-2 diabetes (T2DM) is by far the most common, accounting for approximately 90% of cases worldwide [4]. The acceleration of T2DM prevalence and its associated disease burdens is notably pronounced in low- and middle-income countries (LMICs), where approximately 80% of individuals affected reside [5]. In the Oceania region, the age-standardized incidence rate and mortality rate of diabetes have been observed to be significantly higher than in other parts of the world [6]. A study suggested that the drastic dietary and lifestyle changes introduced by Japan and the U.S. during the colonial era (1930s–1940s) contributed to the rising T2DM prevalence in Pacific Island nations [7]. Therefore, it is essential to adopt a more comprehensive perspective to understand the diabetes epidemic in this region.

In the Republic of Marshall Islands (RMI), T2DM stands as the most critical health issue, with prevalence rates among the highest globally [2]. A national survey conducted in 2018 reported that 27% of individuals aged 18 and older had diabetes, with the prevalence rising to 50% among those over 35 years old. Notably, T2DM accounts for 99.8% of all diabetes cases, and approximately 65% of cases in RMI remain undiagnosed and unaware of their chronic conditions, making it one of the most severe diabetes epidemics worldwide [8]. In 2012, considering the severity of the T2DM burdens, the government issued a national health emergency in response to the epidemic of non-communicable diseases (NCDs) [9]. However, despite efforts to address the issue, the prevalence of T2DM continues to rise, reaching alarming levels.

Given the chronic and progressive nature of T2DM, self-management is an essential part in diabetes care. Diabetes self-management generally encompasses daily behaviors such as healthy eating, physical activity, blood glucose monitoring, medication adherence, and problem-solving to prevent complications [10,11]. The European Association for the Study of Diabetes, and the American Diabetes Association argued that prescription-based medication alone do not control hypoglycemia [11]. Instead, self-management, primarily driven by patients and their families in day-to-day care, plays a critical role in both preventing and managing complications [10]. Continuous engagement in self-management has been shown to be associated with improved blood glucose control, prevention of complications, enhanced well-being, and reduced diabetes-related mortality [12,13]. However, self-management inadequacies have been reported globally because of its complexity, and its barriers often differ in each context [14–16].

In the context of the RMI, inadequate self-management and delayed treatment-seeking behaviors are also recognized. Although the NCD Division of the Ministry of Health and Human Services (MoHHS) has introduced active screening, community health promotion, and set healthcare fees at an affordable $5 per visit [17], a report from Majuro Hospital—the only public healthcare facility in the capital—revealed that 73% of diagnosed diabetes patients did not receive medical treatment necessary for effective T2DM management throughout the year. Previous research conducted in the Marshallese community in Arkansas, U.S., documented that a significant percentage of people tended to ignore or deny the diagnosis, only taking action when the condition became extremely severe. Additionally, this study unveiled that they held severe and peculiar illness perceptions towards diabetes, perceiving it as “inevitable,” “out of our control,” and “death sentence.” Furthermore, it has been documented that there is often a lack of awareness about the benefits of self-management [18].

Among many factors influencing T2DM self-management, the illness perception, along with prerequisite knowledge, is recognized as a crucial factor influencing patients’ disease-coping behavior [19]. Illness perception refers to the implicit and common-sense beliefs that patients hold about their illness, including causal factors, consequences, identity (subjective symptoms), timeline (duration of illness), and controllability [20]. The self-regulation model of illness perception explains that a patient's emotional and cognitive representation of their illness varies depending on the sociocultural context and their unique experiences [21]. The health belief model demonstrates that an individual is more likely to take proper disease-coping action when they perceive a health risk to be serious, feel personally susceptible to it, and believe that the benefits of engaging in preventive measures exceed the associated costs [22]. Illness perception has been linked to the extent of self-management practices in individuals with T2DM, as shown in several studies [23,24]. Moreover, a meta-analysis highlights its pivotal role in shaping patient behavior across a range of diseases, including diabetes [19].

There is a significant gap in the literature regarding inadequate self-management of T2DM within the context of the RMI. Given the severity of the situation, a deeper understanding of patients’ perspectives and the barriers that hinder effective disease-coping actions is essential. This knowledge is crucial for designing targeted interventions to address the ongoing T2DM epidemic. Therefore, we conducted both quantitative surveys and qualitative interviews to achieve two main objectives: (1) identifying the factors associated with T2DM patients’ self-management practices, with a particular focus on the relationship between illness perception and self-management behaviors, as well as the knowledge-action gap; and (2) exploring the social and external backgrounds that shape the current perceptions and self-management practices among T2DM outpatients.

## 2. Materials & methods

### 2.1. Study design

**Fig 1:**
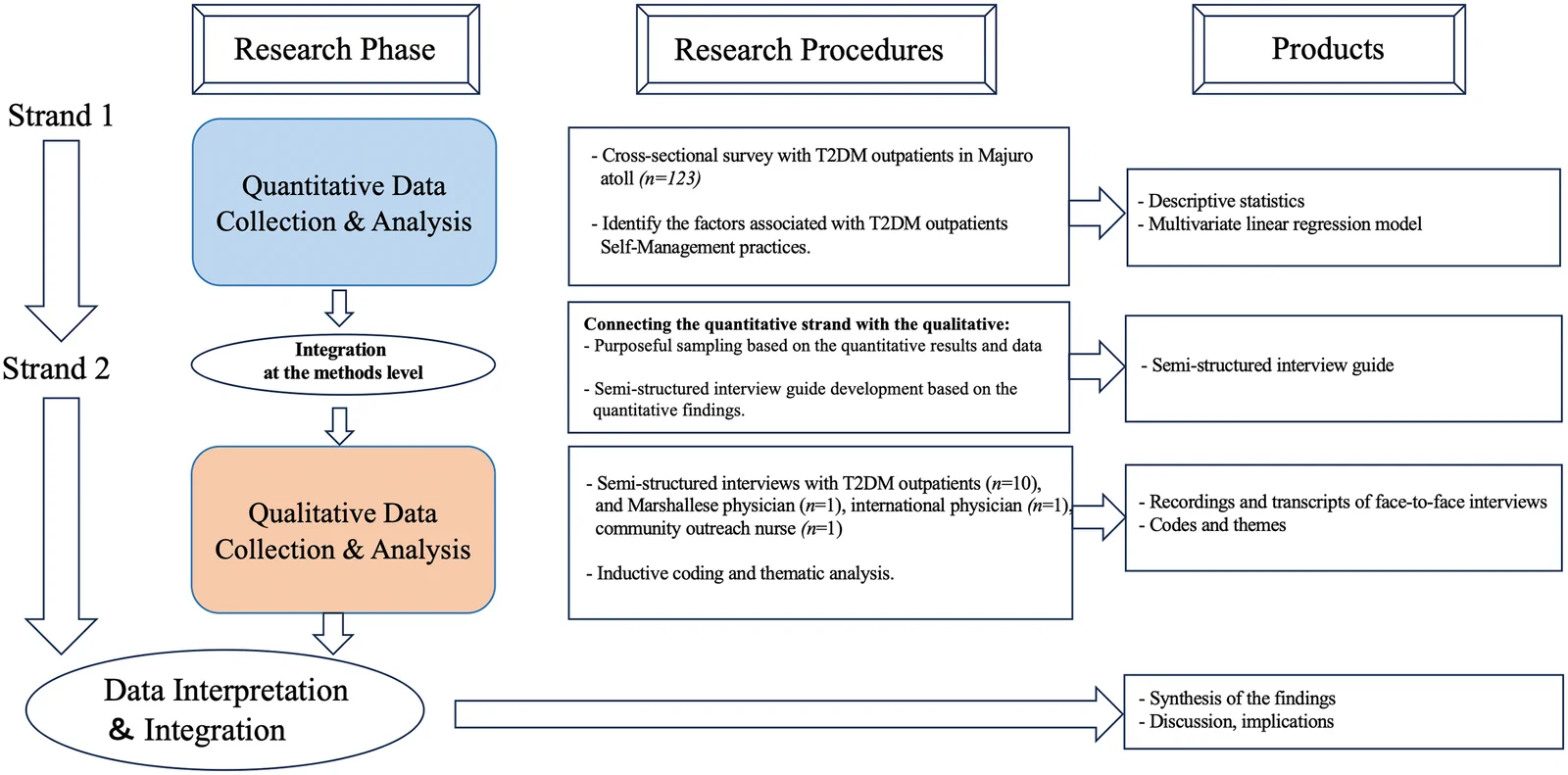
Study flow for the explanatory sequential mixed method design.

Fig 1 describes the flow of this study. We conducted a cross-sectional explanatory sequential mixed-methods study in Majuro Atoll, the capital of the RMI, to achieve the study objectives. In the quantitative phase, Marshallese T2DM outpatients participated in a facilitator-assisted survey designed to identify factors influencing diabetes self-management, drawing on the Health Belief Model and Self-Regulation Model as theoretical frameworks. Following this, semi-structured, in-depth interviews were conducted with a purposively selected subset of survey participants and healthcare providers to provide contextual insights into the quantitative findings. To ensure methodological coherence, integration was implemented at both the methods and reporting levels to strengthen the mixed-methods approach [25].

### 2.2. Participants selection

In the quantitative strand, the Chronic Disease Electronic Management System (CDEMS), managed by the MoHHS since 2010, serves as a registry of diagnosed chronic disease patients capturing both clinic attendees at Majuro Hospital and individuals identified through community-based screening programs, and was used as a dataset to create a sampling frame. As of the end of February 2023, the CDEMS contained information on 3,981 diabetes patients. To ensure the internal validity with respect to the study objectives, inclusion criteria were set as follow: ≧ 18 years old; T2DM outpatients living in Majuro atoll; T2DM patients who identify their ethnicity as Marshallese; diagnosed with T2DM for at least one year. Exclusion criteria included: a hospitalization history within the last three months; impairments in the capacity to provide informed consent due to mental or cognitive disorders. As a result, 2717 Marshallese T2DM outpatients were extracted as the sampling frame. The minimum sample size of 118 was calculated to detect the statistical significance of associations between variables with multiple linear regression analysis [26]. Patients listed at the top of the randomly shuffled sampling frame were approached until the required number was reached.

In the qualitative strand, participants were categorized into two groups: (1) T2DM outpatients who participated in the quantitative strand, and (2) physicians and healthcare providers involved in diabetes care at Majuro Hospital and/or in the community, excluding those not in continuous service in the RMI (e.g., short-term dispatched physicians). Purposive sampling was employed to capture a range of perspectives on diabetes self-management. Among patient participants, selection was informed by DSMQ sum scale scores to ensure inclusion of individuals with contrasting levels of self-management (adequate and inadequate), as well as their potential to provide information-rich accounts related to the qualitative topics. In consultation with local investigators, healthcare providers were selected based on their experience in diabetes care and their likelihood of providing information-rich insights relevant to the study objectives. Participant selection also considered willingness and availability to engage in in-depth interviews. Recruitment was conducted iteratively, with additional participants included as needed to capture emerging perspectives. This process continued until data saturation was reached [27].

### 2.3. Data collection

Written informed consent was obtained prior to commencing data collection. All data were collected from March 17th to May 12th, 2023, by the principal investigator (male, public health background) together with two female Marshallese research assistants, who, although without prior healthcare experience, received training from the first author in research ethics, questionnaire administration, and interview techniques, and were fluent in both English and Marshallese. The data collection process was conducted in either English or Marshallese, depending on the participants’ preference. Quantitative data, except for BMI, were collected by questionnaires through face-to-face facilitator-assisted survey in comfortable settings to avoid random answering. All the documents were translated from English into Marshallese by bilingual speakers and then back translated into English to verify accuracy and consistency. When participants had difficulty interpreting a question, the research team rephrased it for clarity when necessary, and sufficient time was given for responses. The questionnaires consist of five segments, socio-demographic characteristics, clinical factors, T2DM self-management practices, illness perception, and knowledge. To calculate the participant’s BMI, weight, and height were measured with standardized protocols. Participants who provided written consent were approached until all forms were completed or participation was withdrawn. On average, the whole process took 30 minutes to complete. The collected questionnaires were digitized on the same day. Any missing data were identified and supplemented through follow-up, ensuring that no missing data remained in this study.

Qualitative data were collected through face-to-face semi-structured interviews using an interview guide developed based on the results of the quantitative analysis, applied consistently across all interviews but modified to ensure flexibility by allowing additional questions tailored to participants’ attributes and experiences [25]. The guide consisted of seven open-ended core questions, each with supplementary probes to encourage elaboration, covering topics such as barriers to diabetes self-management, perceptions of illness, and experiences with healthcare access. When saturation was achieved, ten T2DM outpatients, and three healthcare providers (one Marshallese physician, one international physician, and one community nurse) all with years of experience in T2DM care, were interviewed. The interview sessions were last on average 48 minutes.

### 2.4. Measures

#### 2.4.1. T2DM self-management practices.

The Diabetes Self-Management Questionnaire (DSMQ), a reliable and internationally validated instrument for assessing self-care activities in patients with diabetes, was used to evaluate self-management adherence in this study. Employing a cut-off score of 6 out of 10 can effectively identify patients at high risk [28]. The questionnaire is composed of sixteen items, fifteen of these items are grouped into four sub-domains including “Glucose Management” (five items. e.g., *“I check my blood sugar levels with care and attention.”*), “Dietary Control” (four items. e.g., *“Occasionally I eat lot of sweets or other foods rich in carbohydrates.”*), “Physical Activity” (three items. e.g., *“I do regular physical activity to achieve optimal blood sugar levels.”*), and “Health-Care Use” (three items. e.g., *“I tend to avoid diabetes-related doctors’ appointment.”*). The last item *“My diabetes self-care is poor”* assessing the subjective evaluation of overall self-care adherence. Each item has a four-point likert scale from one to four. To ensure that a higher score indicates better self-management behavior, 9 items with negative statements in the scoring were reverse scored. The score of each item was added together to form four sub-domains, and the sum of all items including the last item was added to form “Sum Scale”. Each raw sum score was transformed into a scale ranging from 0 to 10, including a decimal point according to the authors’ instructions [28].

#### 2.4.2. Illness perception.

The Brief Illness Perception Questionnaire (B-IPQ) was used to evaluate the patient’s cognitive and emotional representations of T2DM threat [20]. The original version of B-IPQ uses the word “illness”, but in this study the word was replaced to “diabetes” to capture the diabetes-specific perception. The questionnaire consisted of nine items. Five items assessed the cognitive representation of diabetes perception, including perceived consequences, timeline, personal and treatment controllability, and illness identity. Two items assessed emotional representations, including concern and emotional responses. One item assessed participants’ understanding of diabetes. These eight items were rated on a response scale ranging from 0 to 10. Scores for positively worded items were reverse coded during analysis. The total score ranged from 0 to 80, with higher scores indicating a greater perceived threat of diabetes. The final item was an open-ended question that explored respondents’ perceptions of causal factors related to T2DM, with participants asked to list up to three factors they considered most significant.

#### 2.4.3. Diabetes knowledge.

To assess general knowledge about diabetes and self-care, the True/False version of the Revised Diabetes Knowledge Test (DKT2) was used [29]. The questionnaire consisted of 20 items, of which 17 assessed general diabetes-related knowledge and 3 assessed knowledge related to insulin use. Participants who were not using insulin were instructed to skip the last three items. The overall score was calculated as the percentage of correct responses, expressed with a decimal point.

The three items employed in this study are subject to copyright protection and were utilized after directly obtaining permission from the authors.

#### 2.4.4. Sociodemographic characteristics and clinical factors.

Sociodemographic characteristics including age, sex, education level, current marital status, job status, monthly household income, and household size which measure the number of people living together were collected. BMI level, duration of having T2DM (years), family history of T2DM, experience in T2DM self-management education, treatment modality was collected as clinical factors.

### 2.5. Data analysis

All collected quantitative data were analyzed using STATA version 17.0 (StataCorp LLC, College Station, TX, USA). Descriptive statistics, encompassing mean, standard deviation (*SD*), median, and frequencies, were calculated for all collected variables. To identify the factors influencing diabetes self-management, two step analyses were undertaken. First, simple linear regression was conducted to examine the crude associations between independent variables and the DSMQ sum scale. For categorical independent variables, dummy variables were created, with reference categories designated using the b. prefix in Stata. This allowed comparison between each category and the designated reference group in the regression models. Then, variables with p-value < 0.10 were included in the subsequent multiple regression model to identify the independent predictors of self-management practices. Backward stepwise multiple regression was applied to determine the best-fit model. In addition, the likelihood ratio test (LRT) was conducted to refine model selection and assess potential effect modification by comparing models. If the p-value of the LRT was < 0.05, the more complex model was considered the better fit [30]. The variance inflation factor technique (VIF) was employed to address multicollinearity in the final model with the cut-off value of 5 [31].

In the qualitative strand, the audio recorded interviews were transcribed into English transcripts. Interviews conducted in Marshallese were translated into English transcripts by a bilingual research assistant, which were then verified by another Marshallese native speaker who is fluent in English. The transcripts were reviewed three times by the principal investigator to become acquainted with the contents. Following this, thematic analysis was conducted using an inductive approach, allowing key themes to emerge from the data. Data credibility and confirmability were enhanced through team-based discussions during coding and interpretation to ensure that codes and themes accurately reflected the data. In line with our explanatory sequential design, the qualitative themes and patterns identified were subsequently utilized to contextualize and interpret the quantitative results. Data saturation was achieved at the completion of the 13 interviews.

### 2.6. Ethical approval

Ethical approval was obtained from the Institutional Review Board of School of Tropical Medicine and Global Health, Nagasaki University (No.: NU_TMGH_2022_237_1) and the Medical Institutional Review Board (MIRB) of the RMI. The MoHHS of the RMI granted approval for accessing CDEMS and patients’ data at the Majuro hospital solely for the essential elements required to conduct the study.

### 2.7. Inclusivity in global research

Additional information regarding the ethical, cultural, and scientific considerations specific to inclusivity in global research is included in the Supporting Information (S1 Checklist).

## 3 Results

### 3.1. Quantitative results

Of the 241 individuals approached, 123 met the study criteria and voluntarily participated. Among the 118 who did not participate, reasons included emigration to the U.S. (*n* = 35), being unreachable (*n* = 33), death (*n* = 26), relocation to outer islands (*n* = 18), and not meeting the inclusion criteria (n = 6), who were misclassified as Marshallese in the dataset but identified as other ethnicities when approached, and who were not able to give informed consent due to cognitive impairments. No one declined to participate.

#### 3.1.1. Descriptive analysis of sociodemographic, clinical factors, illness perception and diabetes knowledge.

Table 1 presents the results of the descriptive analysis of independent variables. The study included 123 T2DM patients, with ages ranging from 33 to 86 years (*Mean* = 57.5, *SD* = 10.6). The gender distribution was slightly skewed toward females (52.8%). Regarding employment status, 34.1% of participants were unemployed, while 30.1% were employed by the government, and 47.2% had a monthly household income of less than USD 499, the most common income group among participants. The mean BMI value was 30.3 kg/m^2^ (*SD* = 8), with 36.6% classified as overweight and 40.6% as obese (Class I–III). The duration of diabetes varied widely, and the most common duration was diagnosis within the past five years (43.1%). In terms of treatment modality, 46.3% of participants used tablet medication, 4.9% relied solely on Marshallese traditional medicine, and 19.5% did not use any medication at the time of data collection. Moreover, 63.4% of participants had never participated in a diabetes education class.

**Table 1:** Descriptive statistics and simple linear regression analysis of factors associated with DSMQ sum scale (*n* = 123).

Variables	Frequency (%)	Mean (*SD*)	Median	*p*-value
Age (years)		57.5 (10.6)	57	0.005**
Sex				0.155
Male	58 (47.2%)			
Female	65 (52.8%)			
Education level				0.918
None-Primary	8 (6.5%)			
Middle	20 (16.3%)			
Secondary	67 (54.4%)			
Post-secondary	14 (11.4%)			
Tertiary and above	13 (11.4%)			
Job status				0.103
None	42 (34.1%)			
Governmental Worker	37 (30.1%)			
Self-employee	10 (8.1%)			
Private company	19 (15.5%)			
Retired	15 (12.2%)			
Household size		6.7 (3.7)	6	0.588
Monthly household income ($)				0.421
1-499	58 (47.2%)			
500-999	21 (17.1%)			
1000-1499	19 (15.4%)			
1500-1999	5 (4.1%)			
2000-2499	3 (2.4%)			
2500-	17 (13.8%)			
Marital status				0.034*
Single	11 (8.9%)			
Married	83 (67.5%)			
Widowed	10 (8.1%)			
Other	19 (15.5%)			
BMI				0.138
Underweight	1 (0.8%)			
Normal	27 (22%)			
Overweight	45 (36.6%)			
ObesityⅠ	25 (20.3%)			
ObesityⅡ	15 (12.2%)			
ObesityⅢ	10 (8.1%)			
Duration of diabetes				0.316
<5 years	53 (43.1%)			
5-9 years	16 (13%)			
10-14 years	21 (17.1%)			
≧15 years	33 (26.8%)			
Treatment modality				<0.001***
Tablet	57 (46.3%)			
Insulin	23 (18.7%)			
Traditional medication	6 (4.9%)			
Combination^†1^	13 (10.6%)			
None	24 (19.5%)			
Family history of T2DM				0.044*
Parents	63 (51.2%)			
Grandparents	3 (2.4%)			
Both	37 (30.1%)			
None	20 (16.3%)			
Attended diabetes class ever				0.009**
Yes	42(34.2%)			
Never	81(65.8%)			
Illness Perception (B-IPQ)		40.3 (12.9)	40	0.001***
Knowledge (DKT2)		42.4 (11.8)	44.4	0.054

*p < .05　** *p* < .01 *** *p* < .001

†1Combination of western medications (tablet or insulin) with Marshallese traditional medication.

**Fig 2:**
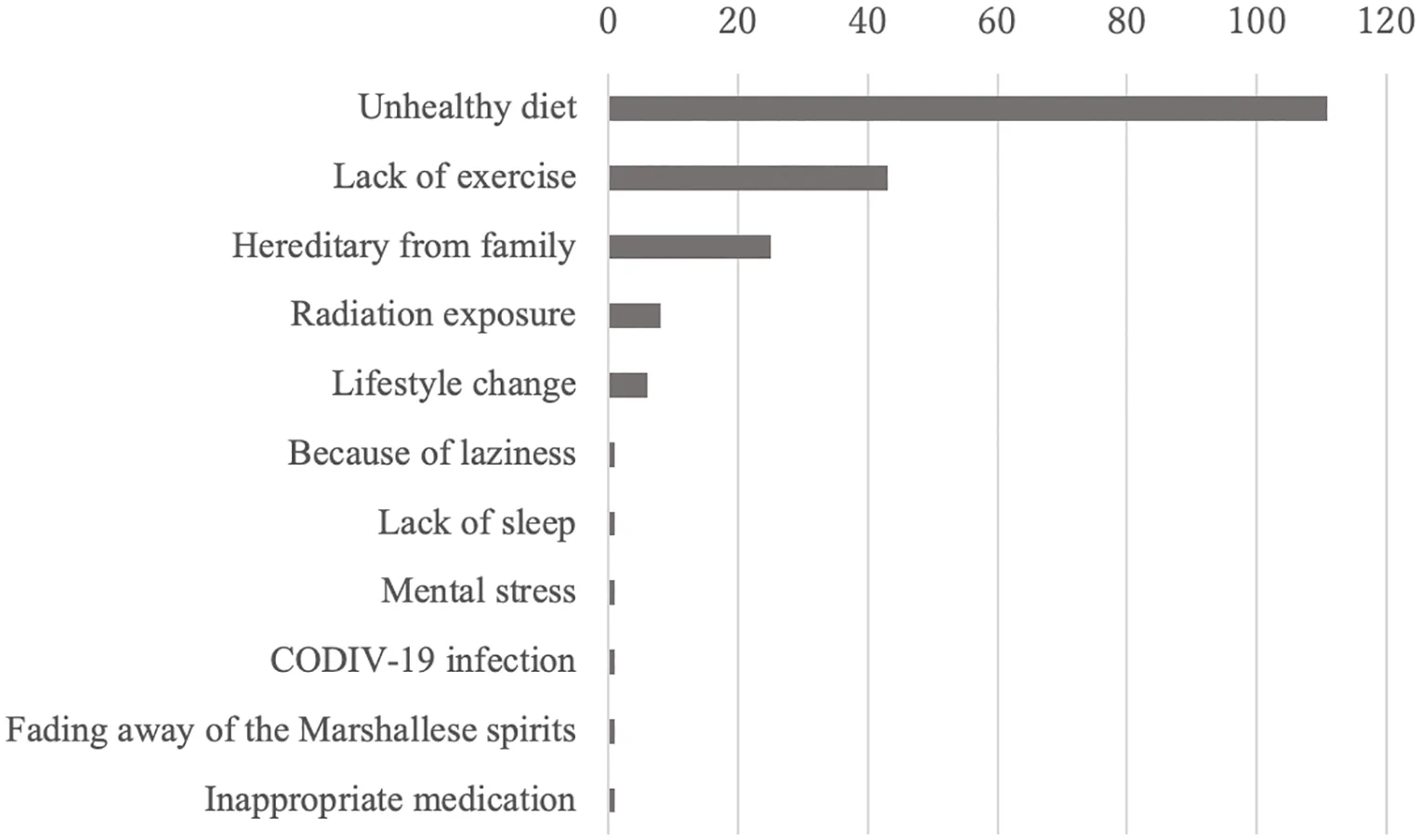
Patients’ self-perception of the causal items of diabetes.

Fig 2 shows the patients’ self-perceived causes of diabetes, derived from the open-ended item of the B-IPQ. The majority of participants (90.2%) responded that “Unhealthy diet” (*e.g., diet change, unhealthy food, sweet beverage*) was cause of diabetes, 43 (35%) responded “Lack of exercise”, and 25 (20.3%) responded “Hereditary from family” (*e.g., Because my family also has or had, Inheritance, Gene*), and 8 (6.5%) responded “Radiation”.

#### 3.1.2. Descriptive analysis of DSMQ scores.

**Fig 3:**
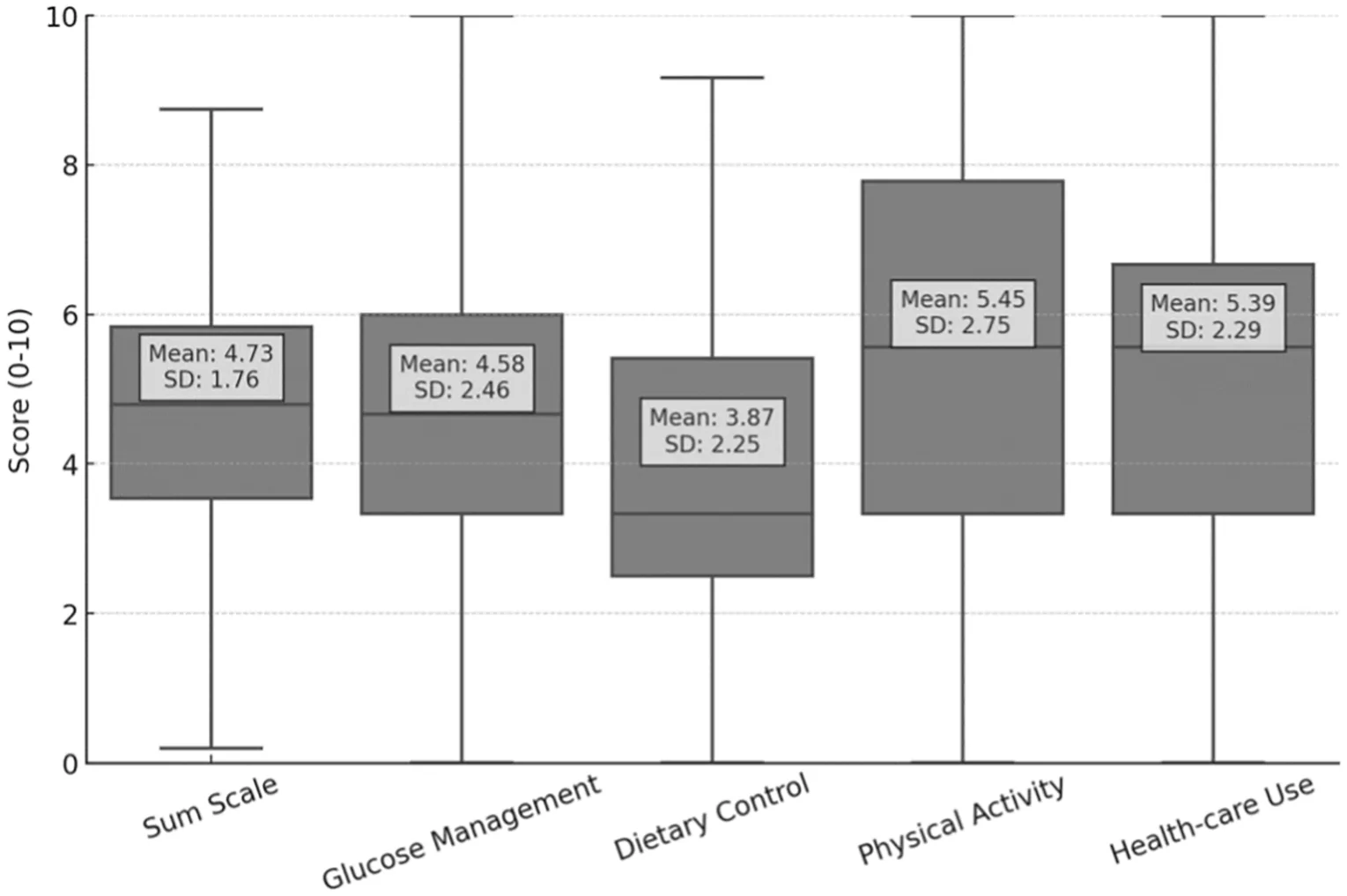
Distribution of DSMQ scores (*n* = 123).

Fig 3 shows the results of a descriptive analysis of DSMQ scores. The data distribution was assessed using histograms and the Shapiro-Wilk test, both of which confirmed normality. The self-management sum score widely ranged from 0.2 to 8.75 (*Mean* = 4.73, *SD* = 1.76). Using the cut-off score of 6, 75.6% of participants were classified as “Inadequate control” and were identified as being in the high-risk group [28]. Among the sub-domains, notably, “Dietary Control” showed the lowest mean score (*Mean* = 3.87, *SD* = 2.25), indicating a particularly low level of adherence.

#### 3.1.3. Factors associated with DSMQ sum scale*.*

Table 1 also presents the results of simple linear regression analysis between independent variables and DSMQ sum scale. Illness perception was negatively associated with DSMQ sum scale (*B* = -0.039, *p* = 0.001), indicating that higher illness perception scores were linked to poorer self-management. Both attending diabetes education classes (*p* = 0.009) and treatment modality as a whole (*p* < 0.001) were significantly associated with DSMQ sum scale, which is expected given that structured education programs and appropriate treatment are key components of diabetes self-management. Diabetes knowledge did not reach statistical significance (*p* = 0.054), despite being commonly recognized as a key factor. In addition, age (*p* = 0.005), family history of diabetes (*p* = 0.044), and marital status (*p* = 0.034) were also significantly associated with DSMQ sum scale. Variables with p-values below 0.10 were included in the subsequent multiple regression model to identify independent predictors of DSMQ sum scale.

#### 3.1.4. Result of multiple linear regression analysis for self-management practices.

Table 2 presents the results of the multiple linear regression analysis for DSMQ sum scale. Backward stepwise multiple regression was applied, initially including variables with p < 0.10 in the simple linear regression. However, age, family history, and marital status were removed through stepwise selection and the likelihood ratio test (LRT) due to non-significance in the final model (*p* > 0.05). After adjusting for other covariates, illness perception remained a significant negative predictor of self-management (*B* = -0.035, *p* = 0.001). Both attending diabetes education classes (*B* = 0.658, *p* = 0.025) and treatment modality as a whole (*p* < 0.001) were significantly associated with DSMQ sum scale, with patients using a combination of traditional and modern treatments showing the highest self-management scores (*B* = 2.771, *p* < 0.001). Knowledge did not demonstrate a statistically significant association with DSMQ sum scale (*B* = 0.019, *p* = 0.100), but it was retained in the final model due to its theoretical importance. The final model explained 33.6% of the variance in DSMQ sum scale (Adjusted R^2^ = 0.296). No substantial multicollinearity was observed (VIF = 1.33).

**Table 2:** Multiple linear regression for DSMQ sum scaleindepentent variable.

	Unadjusted coeficient (*B*)	95% Confidence Interval		Standardized coeficient (*β*)	Std.Err.	*P*-value
		Lower	Upper			
Illness Perception	-0.035	-0.056	-0.014	-0.258	0.011	0.001***
Diabetes Knowledge	0.019	-0.004	0.043	0.130	0.012	0.100
Attended Diabetes Class ever						0.025*†
-Never	ref.					
-Yes	0.658	0.086	1.23	0.178	0.289	0.025*
Treatment Modality						<0.001***†
-None	ref.					
-Insulin	1.256	0.385	2.128	0.279	0.439	0.005***
-Tablet	1.169	0.457	1.882	0.333	0.359	0.002***
-Traditional Medicine	0.277	-1.068	1.624	0.034	0.679	0.683
-Combination	2.771	1.758	3.784	0.487	0.512	<0.001***
-Constant(Intercept)	4.012	2.573	5.45	-	0.726	<0.001***

Model Fit: R^2^ = 0.34, Adjusted R^2^ = 0.29, F(7,115) = 8/31, *p* < 0.001.

^†^ The overall p-values for categorical variables obtained using Wald test.

*p < .05 ** *p* < .01 *** *p* < .001.

### 3.2. Integration of quantitative and qualitative strands

Based on the results of the quantitative analysis, two key qualitative questions emerged as focal points for further exploration: (1) How do factors and individual experiences shape and influence illness perception in T2DM? (2) What factors contribute to the gap between knowledge and self-management practices in T2DM? Understanding these contextual influences is crucial to developing tailored and effective interventions in the RMI [32]. To address these questions, a semi-structured interview guide was developed, and participants were purposively selected from both the quantitative survey respondents and healthcare providers [25].

### 3.3. Qualitative results

A total of 13 informants participated in face-to-face interviews, including ten T2DM outpatients, one Marshallese physician, one international physician, and one Marshallese outreach nurse. To ensure diverse perspectives among T2DM outpatients, five participants were purposively selected from both the “Inadequate Control Group” (DSMQ sum scale < 6) and the “Adequate Control Group” (DSMQ sum scale ≧ 6), considering their DSMQ scores. Table 3 presents age, gender, and the primary questionnaire scores of the informants. The physicians and the nurse were selected based on their extensive experience in providing diabetes care to Marshallese T2DM patients. Three key themes emerged from the thematic analysis and provided contextual insights into participants’ illness perceptions and self-management practices: *dysfunctional healthcare service delivery*, *diabetes fatalism*, and *food deserts*. Each theme is illustrated with annotated extracts, including the informants’ age group, gender, and their control status based on the DSMQ score or occupation.

**Table 3:** Questionnaire scores of semi-structured interview participants.

Informants	DSMQ Sum Scale	B-IPQ Score	DKT2 Score (%)
*Inadequate control group (DSMQ Sum Scale<6)*			
30s, Male	4.38	55	50
40s, Male	3.33	50	45
60s, Male 1	4.79	52	33.3
60s, Male 2	5	13	44.4
60s, Female	1.04	49	55.6
*Adequate control group (DSMQ*≧6)			
50s, Male	8.75	51	66.7
50s, Female	8.1	18	55
60s, Male	7.29	17	50
70s, Male	6.46	39	50
70s, Female	6.88	15	44.4

#### 3.3.1. Dysfunctional healthcare service delivery.

Participants from the qualitative interviews highlighted several dysfunctions in T2DM healthcare services, which were categorized into four sub-themes: “Language barrier,” “Insufficient explanation from doctors,” “Miscommunication and misunderstanding,” and “Limited availability of medical resources.”

[Language barrier]

Both patients and physicians highlighted language barriers as a major challenge in medical settings, particularly due to the RMI's heavy reliance on foreign doctors. Although English is an official language in the RMI, many individuals, especially the elderly and unemployed, have limited proficiency. Thematic analysis revealed that language barriers significantly hinder communication, preventing patients from fully understanding T2DM management.

*“Most of doctors are outsiders like from Philippines or from Taiwan or from even America, India or Fiji. When you go to hospital, and you don't understand English. It is one of the obstacles there.” (30s Male, Inadequate control)*

*“Factor that we've seen I guess it is language barrier. Some might not really understand what the doctors said, especially the foreign doctors.” (30s, Marshallese Doctor)*

*“We can’t express our feeling if you can't speak English. And I think we need someone to be there as a translator.” (50s Male, Adequate control)*

[Insufficient explanation from doctors]

Many T2DM participants reported that physicians primarily prescribed medications without providing sufficient explanations of test results or essential guidance on T2DM management.

*“They don't give you like... something like diet plan or something for the patient. They just talk. Like, you take your medicine, just provide medicine. They give me the same medication again and again every time.” (40s Male, Inadequate Control)*

“They don’t give us the proper and correct information of where, when, and how to see our doctors to check our sugar level.” (60s Male, Poor control)

*“Rush communication. Rush medications. I've seen it in the hospital many times.”* (30s Male poor control)

[Miscommunication and misunderstanding]

The qualitative analysis revealed significant miscommunication and misunderstanding between patients and physicians regarding diabetes medication. Findings indicated that some patients mistakenly believed that diabetes was caused by taking medications such as metformin and insulin. This misconception stemmed from multiple factors, including the asymptomatic nature of early-stage diabetes, physical discomfort and blood glucose fluctuations after starting medication, and physician advice that inadvertently reinforced distrust toward T2DM treatments.

*(Interviewer: Why did you stop taking medications?)“When I take my medication, my sugar level went up to 300. That is why I stopped.” (60s female, Inadequate control)*

*“The Marshallese doctor told to my wife, “I will not recommend you take any diabetes medications. Because if you take the Insulin or Metformin. They're not good for your health and they're going to kill you. They're going to kill your nerves.” (30s Male, Inadequate control)*

*“What we've seen a lot is people just throwing away their metformin and not taking it when we prescribe. They think it's the medicine that causes diabetes because there's been misinformed. Because some of the doctors would tell them that if they're taking them off their kidneys injured more. But then it's mostly because diabetes or their hypertension has caused chronic kidney disease. So, they think it's the medicine that causes it.” (30s, Marshallese doctor)*

[Limited availability of medical resources]

Both patients and healthcare providers highlighted persistent shortages of essential medical supplies as a major barrier to diabetes management. The limited availability of blood glucose monitoring materials, insulin, and other essential medications further compounded the challenges of accessing adequate care in the RMI.

*“They ran out of insulin. It’s also I went there yesterday, they said no insulin at the hospital. It’s unstable. I don’t think they have enough money to buy medical service stuffs.” (70s male, Adequate control)*

*“When I repeated the same question, how I can I check my sugar level? The nurse mentioned that hospital ran out of supplies.” (60s Male 1, Inadequate control)*

*“Our supply of lab works like HbA1C, we usually run out.” (30s, Marshallese doctor)*

#### 3.3.2. Diabetes fatalism.

Consistent with the quantitative findings, the qualitative analysis revealed that many participants perceive diabetes as an inherited and inescapable condition. In the quantitative study, 83.7% of participants reported a family history of T2DM. While Fig 2 highlights modifiable lifestyle factors, such as diet and lack of exercise, as primary contributors to T2DM, the qualitative findings suggest that some participants attribute their condition to fate rather than personal choices. This fatalistic perception reinforces a sense of powerlessness, potentially discouraging proactive self-management efforts.

*“The mentality over here the things that usually your grandparents or parents have diabetes, so if you’re Marshallese, you're going to get it someday.” (50s Male, Inadequate control)*

*“We learned from our doctors here. Once you have a family diabetic history, it's easier you to get affected. If you have a family history, diabetes, it's easier for you to get a diabetes. That's the way everybody thinking.” (30s Male, Inadequate control)*

*“I guess that (T2DM Family history) is why many people think that diabetes is inevitable disease or out of control.” (40s Male, Inadequate control)*

#### 3.3.3. Food deserts.

As the quantitative data indicated, “Dietary Control” had the lowest mean score among the DSMQ subdomains, despite over 90% of participants recognizing diet as a major contributor to diabetes. The qualitative findings highlighted substantial barriers to accessing nutritious foods, particularly vegetables and fruits, which are predominantly imported. With globalization and shifting food systems, imported processed foods have increasingly replaced traditional dietary staples, limiting access to healthier options. Given the legal minimum wage of $2 in the RMI (Mywage.org, 2025), the high prices of these imported nutriture foods are disproportionate and unaffordable for much of the population. Moreover, they are only available in a limited number of supermarkets and remain largely inaccessible in smaller local stores. Even physicians providing dietary guidance find it difficult to recommend healthier choices when nutritious foods are financially and logistically out of reach for most patients.

*“Eating rice with any healthy ingredients, it sounds out of my league. Whenever I want to eat healthy food like vegetables my salary does not cover it.” (60s Male 1, Inadequate control)*

*“And sometimes they (T2DM patients) say, “It's easy for you to say that (Eat heathy food for your health) doctor. But for us, where is vegetable here? And if we find out them, we don't have money to buy them.” (50s, International doctor)*

*“We try to change their (T2DM patients) diet, but we cannot really push them to switch it, I just say limit the amount of starch food they're taking. Because we know that they cannot afford to buy healthy food.” (30s, Marshallese doctor)*

In such a food constrained environment, informants disclosed that their daily food choices primarily consist of rice, instant noodles, chicken, and processed canned food. As these options are affordable and readily available even though they understood that relying on these choices has a negative impact on their diabetes control. During the interviews, they expressed frustration over the limited availability of healthier alternatives on the island.

*“(The reason of diabetes epidemic) Because of eating unhealthy food, although we know and understand that it is not good for our health. We can’t reach nutrition.” (60s Female, Inadequate control)*

*“Rice, ramen, chicken conned beef. That's what they eat here because they have no other choice.” (50s, International doctor)*

*“Mostly they eat rice. If there's no rice, they say we are hungry, they're starving. No more right food here.” (50s, Outreach nurse)*

## 4. Discussion

To the best of our knowledge, this is the first study conducted in the RMI to explore the barriers and underlying factors contributing to inadequate self-management in T2DM patients. In the quantitative strand, illness perception was identified as a significant negative predictor of diabetes self-management. This suggests that individuals who perceive T2DM as more threatening are less likely to engage in self-management, highlighting the need to explore environmental and contextual factors that shape illness perception. Although diabetes knowledge showed a positive correlation with self-management scores, it did not reach statistical significance. This finding suggests a “know-do gap,” where having knowledge about diabetes management does not necessarily translate into adherence to self-care behaviors. In the qualitative strand, participants revealed substantial barriers beyond individual control, including limited and dysfunctional T2DM healthcare capacity, fatalistic perceptions toward diabetes, and food deserts, all of which hinder self-management and shape illness perception. They also perceived those broader social and structural determinants as contributing to the ongoing T2DM epidemic in the RMI.

One common challenge in mixed methods research is the potential mismatch between quantitative and qualitative findings when integrating data. However, this study employed an explanatory sequential design, ensuring strong methodological integration [25]. The qualitative phase was directly shaped by the quantitative results, guiding the development of qualitative research questions and participant selection. Given this design, direct comparison between quantitative and qualitative results is not the objective; rather, the qualitative findings were used to contextualize and deepen the understanding of the quantitative results, as described in the methods section [33].

### 4.1. The influence of illness perception on self-management and its formative context

The severe illness perception of T2DM has previously been documented within the Marshallese community in Arkansas [18], and this study is the first to systematically demonstrated its significant negative impact on self-management practices. The negative influence of illness perception aligns with previous studies [24,34], which suggest that perceiving T2DM as a severe and uncontrollable disease contributes to poorer self-management. Furthermore, illness perception has been shown to extend beyond self-management, significantly impacting HbA1C control and overall T2DM prognosis [35–37]. In light of the health brief model, heightened fear and a sense of helplessness may lead individuals to disengage from proactive self-management behaviors, ultimately reinforcing poor health outcomes [22].

Causal beliefs play a crucial role in shaping illness perception. Quantitative data revealed that over 90% of participants identified diet as a key factor in diabetes onset, indicating widespread recognition of its importance. However, dietary control exhibited significantly lower average scores than other DSMQ sub-domains, suggesting that participants faced substantial difficulties in managing their diet. The qualitative findings highlighted “food desert” conditions as a major barrier to dietary management and a potential contributor to illness perception [34]. Food insecurity is a well-documented issue in the RMI. According to the Food and Agriculture Organization (FAO), approximately 30% of households experience moderate to severe levels of food insecurity. Regarding dietary quality, a recent FAO report found that the average calorie intake in Majuro is 3,001 kcal per capita per day, with 60% of calories coming from unhealthy foods such as rice, sugar, and canned meat [38]. Additionally, 94.5% of adults fail to meet the World Health Organization’s (WHO) recommended daily vegetable intake of 400g [8]. Beyond its impact on diet, food insecurity has been linked to reduced medication adherence and lower self-efficacy in disease management [39]. Although direct evidence from LMICs remains sparse, a study in the U.S. further demonstrated, through path analysis, that food insecurity indirectly and directly affects multiple self-care behaviors—including medication adherence, physical activity, and blood glucose monitoring—by increasing stress and diabetes-related distress [40]. Given these challenges, it is plausible that food insecurity reinforces a fatalistic illness perception among individuals with T2DM. Despite recognizing the importance of diet, many participants may feel unable to act upon this knowledge due to financial and environmental constraints. These findings suggest that addressing food accessibility issues is essential not only for improving dietary behaviors but also for reshaping illness perceptions and enhancing self-management efforts [40].

The fatalistic viewpoints on T2DM may reinforce illness perception and hinder self-management efforts. Both quantitative and qualitative results indicated that participants held a fatalistic perspective on diabetes, attributing its occurrence to family history and genetics in addition to lifestyle-related factors. While genetic predisposition and family history undoubtedly contribute to the risk of developing T2DM [41], extensive evidence demonstrates that T2DM can largely be prevented through proper weight management, dietary adjustments, and regular physical activity (WHO, 2024). Fatalism, defined as “a doctrine that events are fixed in advance so that human beings are powerless to change them” (Merriam Webster, 2025), has been previously reported among the Marshallese community in Arkansas [18]. In that study, a participant expressed this sentiment by stating, “*(diabetes is) our community’s curse*”. Similarly, studies in the U.S. and Mexico have shown that heightened fatalism regarding T2DM is associated with poor HbA1c control and inadequate self-care behaviors [42,43]. Given these findings, it is likely that the perception of high diabetes prevalence within households and communities in the RMI further reinforces internal barriers, contributing to a sense of inevitability and discouraging proactive self-management behaviors. Addressing this deeply ingrained fatalistic belief system may be essential for promoting more effective diabetes management strategies.

### 4.2. Barriers contributing to the “know-do gap”

The current study identified substantial barriers contributing to a significant know-do gap in T2DM self-management. The quantitative results showed that possessing a deeper knowledge does not necessarily translate into improved self-management practices. Previous studies have reported varying associations between diabetes knowledge and self-management behaviors, highlighting the complexity of this relationship [44,45]. While knowledge is essential, intrinsic motivation and external conditions play crucial roles in determining patients’ ability to implement necessary self-care behaviors [22,46]. The qualitative findings further illustrated the external barriers that hinder effective self-management. Participants described persistent shortages of essential medical supplies, language barriers, miscommunication with healthcare providers, and limited access to healthy food. These barriers, largely beyond individual control, contribute to the know-do gap by limiting patients’ capacity to act on their knowledge [47]. Based on the Explanatory Model and Self-Regulation Model, the feedback loop from the consequences of disease-coping actions is reflected in both cognitive (knowledge) and emotional (illness perception) representations, which subsequently shape future self-management behaviors [21,48]. In the RMI, where both healthcare personnel and diabetes care resources are scarce, learned helplessness due to resource unavailability and healthcare system dissatisfaction may discourage further engagement in self-management.

Furthermore, treatment modality was found to be significantly associated with self-management behaviors. Among various treatment approaches, participants using a combination of traditional Marshallese medicine and modern treatments exhibited the highest self-management scores (*p < 0.001*). This suggests that integrating traditional beliefs and practices into diabetes care may facilitate adherence and engagement in self-care. Prior studies have shown that culturally tailored interventions incorporating traditional healing practices can enhance treatment adherence and patient satisfaction [49,50]. Similarly, a systematic review on Aboriginal populations in Australia highlighted that cultural identity and self-determination play a critical role in fostering health and well-being, emphasizing that maintaining a strong connection to culture supports health behaviors and engagement with medical care [51]. These findings suggest that in the RMI, where traditional healing practices remain deeply rooted, integrating culturally relevant approaches into diabetes management may enhance patient engagement and improve self-management outcomes.

Previous systematic reviews and meta-analyses have demonstrated that patient education alone does not significantly improve T2DM outcomes, including HbA1C control [52]. Instead, they emphasize the significance of interventions targeting environmental factors that influence behavior [53]. The findings underscore the importance of addressing external barriers that contribute to the know-do gap in T2DM self-management. Specifically, improvements in healthcare accessibility and food security are essential to bridging this gap and strengthening diabetes self-management in the RMI.

### 4.3. Strengths and limitations

This study is subject to several limitations that should be considered when interpreting the results. First, given its cross-sectional design, causal relationships cannot be established, and potential confounding factors may not be fully accounted for. Secondly, the study is subject to sampling bias, as the CDEMS dataset used for participant recruitment does not encompass all T2DM patients in Majuro. This means that individuals who have not undergone diabetes screening or received a diagnosis were not included, potentially underrepresenting undiagnosed cases. Additionally, the reliance on self-reported data introduces the possibility of recall bias and social desirability bias, which may have influenced the accuracy of responses. Regarding generalizability, variations in living conditions, lifestyle, and healthcare access across different atolls suggest that these findings may not be directly applicable to other regions of the RMI. Future research should explore self-management behaviors in a broader population across multiple settings. Despite these limitations, this study represents the first comprehensive investigation into the factors influencing T2DM self-management in the RMI. The findings offer a crucial baseline for future research and can inform tailored interventions aimed at improving diabetes management and health outcomes in this high-risk population.

### 4.4. Recommendations

The findings of this study suggest various implications for addressing existing barriers to T2DM self-management in the RMI. First, healthcare providers must recognize that patients’ perceptions of T2DM play a crucial role in shaping their self-management behaviors and should be integrated into patient-centered care strategies. It is crucial to foster effective and supportive communication with patients by utilizing culturally appropriate educational materials, interpreters, and patient-centered counseling methods. Additionally, implementing culturally adapted and action-oriented education programs that move beyond passive knowledge dissemination to include skill-based learning, behavioral reinforcement, and peer support models is essential [49,54]. Providing patients with a sense of autonomy and empowerment, while ensuring comprehensive explanations of their medical condition, is key to improving self-management. Secondly, there is a pressing need to enhance the healthcare services in Marshallese by effectively overcoming the language barrier and addressing the immediate demands of patients. Increasing the presence of Marshallese doctors and nurses through government led training and recruitment programs, alongside securing a stable supply of essential medical resources, is essential. Finally, policymakers should implement pricing regulations to make healthy foods more affordable and accessible, while also introducing taxation on unhealthy products such as sugary foods, tobacco, and alcohol. These policy measures align with WHO's ‘Best Buys’ recommendations, which have been successfully implemented in multiple countries, demonstrating their effectiveness in reducing non-communicable diseases and improving public health [55–57].

## 5. Conclusion

This study is the first to comprehensively examine the barriers to T2DM self-management in the RMI using a mixed methods approach. The findings highlight the detrimental impact of heightened illness perception, the persistence of a knowledge-action gap, and structural barriers beyond individual control. To address these challenges, strengthening patient-provider communication, ensuring a stable supply of diabetes medications, and implementing multi-sectoral interventions that integrate culturally adapted education and environmental policy changes are crucial for improving self-management and mitigating the T2DM epidemic in the RMI.

## Supporting information

S1 Checklist Inclusivity in global research questionnaire. Completed PLOS questionnaire on ethical, cultural, and scientific considerations related to inclusivity in global research
(DOCX)

S1 Data De-identified quantitative dataset. De-identified participant-level quantitative dataset underlying the quantitative findings reported in this study.
(XLSX)
